# Polycyclic aromatic hydrocarbons and their oxygenated derivatives in urban aerosol: levels, chemical profiles, and contribution to PM_2.5_ oxidative potential

**DOI:** 10.1007/s11356-021-16858-z

**Published:** 2022-03-17

**Authors:** Maria Chiara Pietrogrande, Dimitri Bacco, Giorgia Demaria, Mara Russo, Fabiana Scotto, Arianna Trentini

**Affiliations:** 1 Department of Chemical, Pharmaceutical and Agricultural Sciences, University of Ferrara, Via Fossato di Mortara 17/19 - 44121, Ferrara, Italy; 2 Emilia Romagna Regional Agency for Prevention, Environment and Energy, ARPAE, Via Po 5 - 40139, Bologna, Italy

**Keywords:** PM_2.5_, Polycyclic aromatic hydrocarbons, Oxygenated PAHs, Oxidative potential, Redox-active quinones

## Abstract

The concentrations of polycyclic aromatic hydrocarbons (PAHs) and quinones, a subgroup of oxygenated PAHs (oxy-PAHs), were measured in PM_2.5_ samples collected during warm (May–June 2019) and cold (February–March 2020) seasons in the city of Bologna, Italy. Total PAHs concentration was nearly double in winter (6.58 ± 1.03 ng m^−3^) compared with spring (3.16 ± 0.53 ng m^−3^), following the trend of the PM_2.5_ mass concentration. Molecular diagnostic ratios suggested that, together with traffic, biomass burning was the dominant emission source contributing to the peaks of concentration of PM_2.5_ registered in the cold season. Quinone level was constant in both seasons, being 1.44 ± 0.24 ng m^−3^, that may be related to the increased secondary formation during warm season, as confirmed by the higher Σoxy-PAHs/ΣPAHs ratio in spring than in winter. The oxidative potential (OP) of the PM_2.5_ samples was assessed using acellular dithiothreitol (DTT) and ascorbic acid (AA) assays. The obtained responses showed a strong seasonality, with higher volume-normalized (OP_V_) values in winter than in spring, i.e., OP_V_^DTT^: 0.32 ± 0.15 nmol min^−1^ m^−3^ vs. 0.08 ± 0.03 nmol min^−1^ m^−3^ and OP_V_^AA^: 0.72 ± 0.36 nmol min^−1^ m^−3^ vs. 0.28 ± 0.21 nmol min^−1^ m^−3^. Both OP_V_^DTT^ and OP_V_^AA^ responses were significantly associated with total PAHs, as a general descriptor of redox-active PAH derivatives, associated with co-emission from burning sources or secondary atmospheric oxidation of parent PAHs. Otherwise, only winter OP_V_^DTT^ responses showed a significant correlation with total Ʃoxy-PAHs concentration.

## Introduction

Polycyclic aromatic hydrocarbons (PAHs) are popular pervasive harmful pollutants in the atmospheric environment and have been largely studied and regulated in ambient air, as some of them are known human carcinogens (Abdel-Shafy and Mansour 2016; Hao et al. 2018; Slezakova et al. 2013). Moreover, over the last years, attention has been redirected to their derivatives, such as nitrated PAHs (NPAHs) and oxygenated PAHs (oxy-PAHs), as they are probably more toxic compared to parent PAHs, due to higher mutagenicity and carcinogenicity (Alves et al. 2017; Lammel et al. 2020; Li et al. 2019; Niu et al. 2017; Wang, et al. 2018). Oxy-PAHs are present in complex mixtures in the atmosphere, both in gas and particle-bound phases, as they can be likely cogenerated with PAHs with soot from incomplete combustion processes of organic materials or photochemically formed from PAH through homogeneous or heterogeneous photo-oxidation reactions with atmospheric oxidants (such as^**.**^OH,^**.**^NO_3_, and O_3_) (Keyte et al. 2013; Lammel et al. 2020; Walgraeve et al. 2010). Thus, given the continuous nature of the exposure to PAHs and oxy-PAHs bound to PM and the size of the population at risk (Albinet et al. 2007; Alves et al. 2017; Andreou et al. 2009; Kramer et al. 2020; Niu et al. 2017; Slezakova et al. 2013), there is a need for their air monitoring, although it is an analytical challenging task, requiring high cost and time and the use of specialized equipment, e.g., mass spectrometer operating in MS/MS acquisition mode (Nalin et al. 2018; Walgraeve et al. 2010).

Considerable interest to PAHs and oxy-PAHs also derives by the finding that they can react with molecular oxygen, coexisting PM chemicals, and cell components to produce reactive oxygen species (ROS), which have been found crucial mediators of PM toxicity through the oxidative stress mechanism (Chowdhury et al. 2019; Crobeddu et al. 2017; Lyu et al. 2018; Jin et al. 2019). For example, some PAHs can be transformed in biosystems into redox-active quinones, subgroup of oxy-PAHs, which can catalyze the formation of H_2_O_2_ and^.^O^2−^ by transferring electrons from NADPH to oxygen (Li et al. 2019; Squadrito et al. 2001; Tsapakis and Stephanou 2007). Given that the mass loadings of several different oxy-PAHs may be high in the atmosphere, quinones have been found to play a relevant role in producing oxidative stress, accounting up to ∼10% of PM oxidative properties (Charrier and Anastasio 2015; Gao et al. 2020; Jiang et al. 2016). However, the role of PAHs and oxy-PAHs in producing oxidative stress is still poorly understood, due to the complexity of the atmospheric oxidation processes to generate oxy-PAHs and the presence of several multifunctional products in secondary organic aerosol.

With this in mind, in this study, we separately investigated ambient PM_2.5_ samples for the quantification of parent and oxygenated PAHs and the assessment of the oxidative properties with the aim to highlight the potential contribution of PAHs and oxy-PAHs to the capacity of PM_2.5_ to produce ROS.

The study was performed on PM_2.5_ samples collected at an urban background site in the Po Valley (Northern Italy) during spring 2019 and winter 2020. The concentration levels of PM_2.5_-bound PAHs and oxy-PAHs were measured, and their composition profile was investigated in order to give information on their origin and seasonal variation.

Then, the oxidative potential (OP) was measured, as a relevant parameter which describes the ability of PM components to deplete antioxidants in vitro and generate ROS (Bates et al. 2019; Calas et al. 2019; Chowdhury et al. 2019; Molina et al. 2020). Two cell-free assays were used that are simply based on spectroscopic measures of target molecules. One uses the dithiothreitol (DTT) to simulate the PM-catalyzed electron transfer from cellular antioxidants (e.g., NADPH) to O_2_, and the other is based on the ascorbic acid (AA), the most abundant antioxidant in lung fluids, which has a vital role in protecting organism against oxidative stress (Bates et al. 2019; Calas et al. 2019; Crobeddu et al. 2017; Gao et al. 2020; Hellack et al. 2017; Pietrogrande et al. 2019b). Finally, the measured OP^DTT^ and OP^AA^ responses were correlated with different PM_2.5_ chemical components, in order to elucidate the chemical markers mostly associated with ROS production.

## Materials and methods

### Chemicals and materials

Methanol (HPLC grades), cyclohexane (HPLC grades), n-hexane ≥ 99%, and dichloromethane ≥ 99,99% were purchased from E. Merck (USA) and used as received.

Individual standards of each PAH and oxy-PAH were purchased from Supelco (Bellefonte, PA, USA). The 16 US EPA priority PAHs were investigated: naphthalene (NAP), acenaphthylene (ACENY), acenaphthene (ACE), acenaphthylene (AcPy), fluorene (FLU), phenanthrene (PHE), anthracene (ANT), fluoranthene (FLUA), pyrene (PYR), benz(α)anthracene (BaA), chrysene (CRY), benzo(b)fluoranthene (BbF), benzo(k)fluoranthene (BkF), benzo(α)pyrene (BaP), indeno(1,2,3-cd)pyrene (InP), dibenz(a,h)anthracene (DbA), and benzo(ghi)perylene (BghiP). In addition, individual standards of 8 quinones, purchased from Acros Organics (New Jersey, USA), were investigated: 9-fluorenone (9-FLO), xanthone (XAN), 9,10-anthraquinone (9,10-AQ), 7,12-benzo(a)anthracenequinone (7,12-BAQ), 5,12-naphthacenequinone (5,12-NQ), 1,2-naphthoquinone (1,2-NPQ), 1,4-naphthoquinone (1,4-NPQ), and 9,10-phenanthrenequinone (9,10-PNQ).

Individual standard stock solutions were prepared at a concentration of 200 μg mL^−1^ by dissolving pure standards using dichloromethane and acetonitrile as solvents for each PAH and oxy-PAH, respectively. Working solutions (at 500 ng mL^−1^ each) were prepared by dilution with dichloromethane. All solutions were stored at –6 °C.

The internal standard solution (EPA 8270 Semi-volatile Internal Standard Mix) was supplied by Supelco (Bellefonte, PA, USA). It contained 6 deuterated PAHs at the concentration of 200 μg mL^−1^ in dichloromethane, i.e., naphthalene-d8, acenaphthene-d10, phenanthrene-d10, chrysene-d12, and perylene-d12. A diluted concentration of 100 ng mL^−1^ was added to standard solutions, for computing the calibration curves, and to samples, for quantifying the target analytes.

Sodium phosphate (NaH_2_PO_4_, ACS) and disodium hydrogen phosphate (Na_2_HPO_4_) were purchased from Fisher Scientific. The 0.1 M phosphate buffer was prepared at pH 7.4 using ultrapure water (Milli-Q® IQ 7000 water purification system). Then, it was treated with Chelex® 100 sodium form resin (BioRad) to remove any metal contamination.

Solutions of DTT and DTNB (5,5′-Dithiobis(2-nitrobenzoic acid)) (Sigma Aldrich) were prepared in phosphate buffer (10 mM), while solutions of L-ascorbic acid sodium salt (AA) (Sigma Aldrich) were prepared in ultrapure water (10 mM). Aqueous solutions of the reagents are unstable at room temperature and sensible to light; thus, they were preserved in amber glass vials in the dark at − 20 °C.

### Study sites and PM collection

**Fig. 1:**
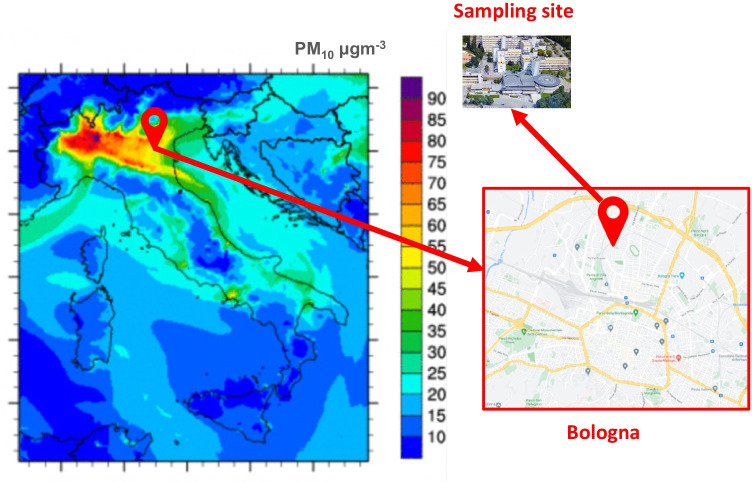
Map of the investigated Bologna area in the Po Valley in the north of the Italian peninsula: the map shows PM_10_ levels measured in different regions (ENEA 2017). The inset shows the ARPAE ER sampling site located in the surrounding of the Bologna historical center (inset of Fig. 1)

Samples were collected in the middle of the city of Bologna (~ 400,000 inhabitants), in the south-eastern part of the Po Valley, in northern Italy. Details on the sampling location are reported in Fig. 1, where a map of the Bologna area gives a view of the surrounding densely populated region (ENEA 2017). Po Valley is regarded as an air pollution hot spot in Europe, with frequent severe PM air pollution events, as a consequence of intense anthropogenic emissions from industry, agriculture, and traffic, as well as high photochemical activity in summertime (Paglione et al. 2021; Ricciardelli et al. 2017). The sampling urban background site was located in the surrounding of the historical center of Bologna (inset of Fig. 1). Traffic and domestic heating during the cold season are the dominant air pollution sources in the area and cause high levels of air pollutants (Pietrogrande et al. 2016).

Four PM_2.5_ samples were simultaneously collected every day during two campaigns: 28 samples from 8 May to 17 June 2019 and 34 filters from 4 February to 9 March 2020. A low volume automatic outdoor sampler (Skypost PM, TCR-TECORA Instruments, Corsico, Milan, Italy) was used, operating at the standard air flow rate of 38.3 L min^−1^ for 24 h to collect an air volume of ≈ 55 m^3^ per day. PM_2.5_ samples were collected on 47-mm-diameter quartz fiber filters provided from Whatman (Whatman® QM-A quartz filters). After sampling, the procedure outlined in European Standard EN 12,341 (https://www.en-standard.eu/csn-en-12341-ambient-air-standard-gravimetric-measurementmethod-for-the-determination-of-the-PM10-or-PM2,5-mass-concentration-of-suspended-particulate-matter) was applied for equilibrating and weighing the collected samples. All details concerning the site and the logistical aspects of the sampling procedure can be found in previous papers (Pietrogrande et al. 2019a; Ricciardelli et al. 2017).

### PAHs and oxy-PAHs analysis

Two PM_2.5_ filters collected in two consecutive days (3 quarters of each filter) were combined and extracted for 30 min in an ultrasonic bath with 15 mL of n-hexane/dichloromethane, 30:70, solvent mixture. The extracts were filtered using a syringe filter (PTFE 25 mm, 0.22 μm, Kinesis) to remove insoluble particles, and then the filtrates were evaporated to dryness in a centrifugal vacuum concentrator (miVac Duo Concentrator, Genevac Ltd, Ipswich, UK) at room temperature. The samples were reconstituted with 500 μL of the extraction solvent mixture, transferred in 2-mL glass vials and dried under gently nitrogen flow. Then, samples were recovered with 100 μL of n-hexane for direct injection into the GC/MS system.

Chromatographic analyses were performed under experimental operative conditions previously validated and widely applied by some of the authors (i.e., Pietrogrande et al. 2011, 2014). A Gas Chromatograph Focus GC (Thermo-Fisher Scientific) was used, coupled with a mass spectrometry detector PolarisQ Ion Trap Mass Spectrometer (Thermo Fisher Scientific, Bellefonte, PA, USA). A Rxi®-5Sil MS capillary column (30 m × 0.25 mm I.D., 0.25 μm film thickness) was purchased from Restek (USA). High purity helium (99.999%) was used as the carrier gas at constant flow rate of 1.5 mL min^−1^. Two microliters of sample was injected in the split/splitless injector maintained at 270 °C (splitless for 5 min). The oven temperature program started at 90 °C and held for 2 min. It was heated up to 320 °C at a rate of 6 °C min^−1^ and finally held for 10 min. The GC/MS interface and ion source temperatures were kept at 280 °C and 250 °C, respectively. The MS analysis was performed in electronic impact mode (EI) on positive mode with an energy of 70 eV. The tandem MS was operated in single reaction monitoring (SRM) mode, by using transition conditions (precursor ion, product ion and collision energy) reported in literature (Nalin et al. 2018; Walgraeve et al. 2010). Data acquisition was performed using Thermo Scientific 1.4 XCalibur program (WestPalm Beach, USA).

The method was validated under the used operative conditions, by evaluating some figures of merit, such as the linear calibration range, limits of detection and quantitation, precision, accuracy, and interferences. The calibration curves were computed using a standard mixture of PAHs and oxy-PAHs (six concentration points ranging from 50 to 3000 ng mL^−1^). Such concentrations in the injected solution corresponded to concentration from 0.002 to 0.11 ng m^−3^ in the sampled air, based on the sampled air volume (55 m^3^ air volume). The analytical limits of detection (LOD) were computed for each individual compound, as the lowest concentration of the compound that can be detected (S/N = 3). Based on the sampled air volumes, LODs ranged from 0.002 to 0.016 ng m^−3^ for investigated PAHs and from 0.005 to 0.029 ng m^−3^ for target oxy-PAHs. Repeatability was evaluated as intra-day precision from five replicate analyses of PAH and oxy-PAHs standard solutions at the medium concentration level of 500 ng mL^−1^ (i.e., 0.018 ng m^−3^ in the sampled air). A good precision was obtained, with a relative standard deviation (RSD %) ranging between 2.7 and 6.2%. The method accuracy was assessed by measuring the recovery on real PM_2.5_ samples spiked with a standard mixture of PAHs and oxy-PAHs (each at concentration of 1000 ng mL^−1^, i.e., 0.037 ng m^−3^ in the sampled air). Satisfying recovery in the 90–72% range was provided for all target compounds. To evaluate interferences in the GC/MS analysis of the real PM_2.5_ samples, method blank was measured on field blank filters submitted to the same extraction and analysis procedure as the PM samples.

### DTT and AA assays for measuring oxidative potential

To measure OP responses, one-quarter of each PM_2.5_ filter was extracted for 15 min in an ultrasonic bath using 10 mL of phosphate buffer. The extracts were then filtered on a regenerate cellulose syringe filter (13 mm, 0.22 μm, Kinesis) to remove the suspended solid particles. Then, 3 mL of the solution was submitted to each OP assays, following the procedure previously used by the authors (Pietrogrande et al. 2019a; Visentin et al. 2016). A constant temperature of 37 °C was kept by using a dry bath. Spectrophotometric measurements were performed in a UV–Vis spectrophotometer (Jasco V-730, JASCO EUROPE s.r.l.) with a 1 cm path length optical cell. Polystyrene and quartz cuvette were used for DTT and AA assays, respectively.

In the DTT assay, 30 μL of the 10 mM DTT solution was added to the sample (i.e., time zero) and the rate of DTT depletion (OP^DTT^) measured as following. At defined times, a 0.50 mL aliquot of the reaction mixture was removed, and the reaction stopped with trichloroacetic acid (0.50 mL of 10%). Then, the remaining DTT is reacted with DTNB to generate DTT-disulfide and 2-nitro-5-thiobenzoic acid (TNB): 50 μL of the DTNB solution (10 mM concentration in phosphate buffer at pH 7.4) was added to each aliquot and well mixed. After 2 min to allow the complete reaction, pH was increased to a value of 8.9, by adding 2.0 mL of Tris–HCl buffer (0.40 M at pH 8.9 with 20 mM of EDTA), to form the mercaptide ion (TNB^2−^), which has a high light absorbance (molar extinction coefficient ε = 14,150 M^−1^ cm^−1^ at 412 nm).

In the AA assay, 30 μL of the 10 mM AA solution was added to the sample (i.e., time zero). Then, the rate of AA depletion (OP^AA^) was followed directly in the spectrophotometric cuvette by measuring the absorption of the ascorbate ion at 265 nm (ε = 14,500 M^−1^ cm^−1^ at pH 7.4) at defined time intervals.

The rate of DTT or AA depletion (nmol min^−1^) was determined by linearly fitting the experimental points of the reagents concentration versus time (5, 10, 15, 25, 40 min). The response of blank filters was determined and subtracted from the data of real PM samples. The obtained OP responses were then normalized both to air collected volume, i.e., volume-normalized OP_V_ (nmol min^−1^ m^−3^) and to PM_2.5_ sampled mass, i.e., mass-normalized OP_m_ (nmol min^−1^ μg^−1^).

### Chemical characterization of ambient PM_2.5_ samples

Other chemical components were analyzed in the laboratories of the Emilia Romagna Regional Agency for Prevention, Environment and Energy in Ravenna (Italy). Details are reported elsewhere (Ricciardelli et al. 2017). Briefly, one filter was directly submitted to thermo-optical transmission analysis to quantify the carbonaceous fraction, i.e., elemental carbon, EC, and organic carbon, OC. A Sunset instrument (Laboratory Inc., Oregon, USA) was used, following the EUSAAR2 thermal protocol, according to the European standard (UNI EN 16,909:2017). Another filter was extracted with 10 mL of Milli-Q water, sonicated for 15 min, filtered on 0.45 μm cellulose acetate filters, and then submitted to the following analyses. Inorganic ions were quantified by Ionic Chromatography: ICS-1000 with IonPac™AS9-HC for anions (Cl^−^, NO_3_^−^, SO_4_^2−^) and ICS-1100 with IonPac™CS12A for cations (K^+^, NH_4_^+^, Ca^2+^) (DIONEX, California, USA). Levoglucosan was quantified using HPLC–MS instrument (HPLC Agilent 1200 series and Triple Quadrupole 6410 equipped with Electrospray Ionization, Agilent Technologies Inc., California, USA) with a ZORBAX amino column. Detection of levoglucosan was achieved by formation of an anhydrosugar acetate adducts [M + CH_3_COO]^−^ in the negative electrospray mode.

One filter was mineralized with 10 mL of a HNO_3_:H_2_O_2_ (8:2) mixture and analyzed for metal quantification using Inductively Coupled Plasma – Mass Spectrometry (7700 ICP-MS, Agilent Technologies Inc., California, USA), following the method reported in UNI EN 14,902:2005.

### Statistical analysis

Non-parametric statistics were used to investigate the dataset, since several parameters lacked normal distribution of the measured values, as checked by Shapiro-Francia and Kolmogorov–Smirnov tests. The Mann–Whitney test was used to single out significant differences (at *p* < 0.05) in the mean measured parameters between spring and winter samples.

Kendall rank correlation analysis was conducted to investigate the statistical dependence between variables, due to its ability in accounting for ties. We graded the strength of the Kendall’s τ coefficient significance as significant (*p* < 0.05) or very significant (*p* < 0.01).

## Results and discussion

### PAH concentrations: seasonal variations and distribution profiles

Fifteen target PAHs were identified and quantified in the study PM_2.5_ samples. The mean and standard deviation values were computed for the concentration of each PAH in spring and winter campaigns, separately (Table 1). The total measured concentrations ranged from 2.25 to 10.14 ng m^−3^, with mean and standard deviation values of 5.03 ± 2.30 ng m^−3^. An insight into the data shows a strong seasonality for nearly all target PAHs, characterized by a very significant (*p* < 0.001) concentration increase in winter compared with spring for FLU, PHE, ANT, FLUA, PYR, CRY, BbF, BkF, InP, and BghiP (indicated by ** in Table 1). The resulting total ΣPAHs concentration was nearly double in winter (6.58 ± 1.03 ng m^−3^) than in spring (3.16 ± 0.53 ng m^−3^). Such a seasonality is common in the Northern Italy (Amato et al. 2016; Belis et al. 2011; Masiol et al. 2013; Pietrogrande et al. 2016; Ricciardelli et al. 2017). It has been explained by additional emission sources in cold season, namely combustion of fossil and biomass fuels for household heating, combined with lower partitioning to vapor compared with the warmer spring. Such a pattern follows the trend of the PM_2.5_ mass concentration with a strong increase in winter (26.7 ± 15.5 μg m^−3^) compared with spring (8.29 ± 3.28 μg m^−3^). Other than to intense local and regional PM source contributions, higher winter levels are also due to the specific meteorology of the Po Valley, with atmospheric stagnant conditions (low mixing height: Hmix ≈ 300 m) and frequent thermal inversions, which induce regional accumulation of pollutants (Amato et al. 2016; Masiol et al. 2013).

**Table 1:** Measured concentrations of each target PAH and oxy-PAH as mean and standard deviation values of the spring and winter campaigns, separately. Bold values indicate statistically significant differences between seasons: *p < 0.01; **p ≤ 0.001. LOD indicates the limit of detection of the analytical procedure

	Spring (ng/m^3^)		Winter (ng/m^3^)	
PAHs	mean	SD	mean	SD
Acenaphthylene (ACENY)			0.18	0.01
Acenaphthene (ACE)	< LOD	-	0.11	0.01
Fluorene (FLU)	0.33**	0.10	0.57**	0.12
Phenanthrene (PHE)	0.42**	0.13	0.23**	0.10
Anthracene (ANT)	0.28**	0.03	0.14**	0.01
Fluoranthene (FLUA)	0.13**	0.02	0.31**	0.18
Pyrene (PYR)	0.15**	0.03	0.34**	0.19
Benzo[a]anthracene (BaA)	< LOD	-	0.71	0.91
Chrysene (CRY)	0.36**	0.05	0.63**	0.28
Benzo[b]fluoranthene (BbF)	0.13**	0.02	0.70**	0.37
Benzo[k]fluoranthene (BkF)	0.49**	0.11	0.37**	0.07
Benzo[a]pyrene (BaP)	0.55	0.01	0.58	0.11
Indeno[1,2,3-c,d]pyrene (InP)	0.60**	0.04	1.22**	0.23
Dibenzo[a,h]anthracene (DbA)	< LOD	-	0.01	0.06
Benzo[g,h,i]perylene (BghiP)	0.22* *	0.07	0.85**	0.36
Oxy-PAHs				
9-Fluorenone (9-FLO)	0.24	0.01	0.23	0.04
Xanthone (XAN)	0.22**	0.02	0.17**	0.01
9,10-Anthraquinone (9,10-AQ)	0.27	0.03	0.25	0.04
7,12-Benzo(a)anthracenequinone (7,12-BAQ)	0.42*	0.02	0.37*	0.06
5,12-Naphthacenequinone (5,12-NQ)	0.48*	0.08	0.39*	0.06

Overall, the PAHs concentrations measured in this work appear broadly consistent with those described in urban atmospheres in other Italian sites, such as in the close city of Florence (traffic and background sites, Martellini et al. 2012; Alves et al. 2017), in the close region Veneto (different sites, Masiol et al. 2013), and in Milan, a megacity in Northetrn Italy (Hakimzadeh et al. 2020; Pietrogrande et al. 2014). In addition, similar values with the same season trend were found in urban atmospheres in Europe (Abdel-Shafy and Mansour 2016; Albinet et al. 2007; Amato et al. 2016), such as in Paris (Ringuet et al. 2012), Athens (Andreou and Rapsomanikis 2009), Oporto (Slezakova et al. 2013), Birmingham (Alam et al. 2014), and Czech Republic (Lammel et al. 2020).

Concerning the PAH distribution profiles, an increasing concentration in particulate phase with molecular weight was observed, with the dominant five and six rings congeners—BaA, BbF, BkF, InP, and BghiP—as commonly seen in PAH datasets (Alves et al. 2017; Li et al. 2019; Martellini et al. 2012). The heavier BbF, BkF, InP, and BghiP have been associated with gasoline-powered vehicles, while lighter PAHs have been found more abundant in diesel exhausts (Abdel-Shafy and Mansour 2016; Hao et al. 2018; Lin et al. 2019; Riccio et al. 2016).

In order to give insight into the main emission sources generating the PM-bound PAHs, some molecular diagnostic ratios were computed, as useful tool to distinguish among different origins (Tobiszewski and Namieśnik 2012; Li et al. 2019). The ratio ANT/(ANT + PHE) indicated that sources related to combustion processes were dominant, as the computed values were always above 0.3 (mean value 0.36 ± 0.05), since the ratio > 0.1 is diagnostic of pyrogenic origin and < 0.1 of petrogenic source. The FLUA/(FLUA + PYR) ratio was computed to distinguish among different combustion fuels, as it assumes a value < 0.4 for petrogenic combustion and > 0.5 for grass, wood, and coal burning. In this study, a value of 0.48 ± 0.02 was computed that suggested a combined contribution of both combustion sources. The InP/(InP + BghiP) ratio can be used for discriminating between petroleum (< 0.5) and grass/wood burning (> 0.5). Thus, the mean value of 0.61 ± 0.05 found in this study indicated that wood burning emission was the dominant source. Higher ratios were found in spring (0.70 ± 0.03) than in winter (0.60 ± 0.03): this is consistent with the enhanced contribution of photochemical reactions, as BghiP has been found to photodegrade faster than InP, so that particle aging shifts the InP/(InP + BghiP) ratio towards high values (Tobiszewski and Namiesnik 2012). Finally, the BaA/(BaA + CRY) ratio was computed to discriminate vehicle emissions between gasoline and diesel exhausts, characterized by values ~ 0.73 and ~ 0.5, respectively. Here, a value of 0.54 ± 0.13 was computed for the winter data, suggesting that PAHs were predominantly emitted by diesel engines.

### Association of PAHs concentrations with other chemical components

Some chemical tracers were quantified in each PM_2.5_ sample to give a general description of PM_2.5_ sources and processes, i.e., elemental and organic carbon, secondary ions (SO_4_^2−^, NO_3_^−^, NH_4_^+^), ions (Cl^−^, K^+^, Ca^2+^), levoglucosan, and transition metals, i.e., Fe, V, Zn, Cd, Pb, Sn, Sb, La, and Mn. The mean and SD values were computed on the whole dataset as well as on the spring and winter values, separately, and are reported in Table 2. By comparing the two seasons, we can observe that several species showed significantly (*p* < 0.005) higher values in winter than in spring, such as nitrate and ammonium ions, EC, OC, levoglucosan, and transition metals (indicated by bold values in Table 2). This is consistent with seasonal trend commonly found in the Northern Italy (Amato et al. 2016; Belis et al. 2011; Masiol et al. 2013; Pietrogrande et al. 2016; Ricciardelli et al. 2017). Otherwise, the most abundant iron showed nearly constant values along the year (57.1 ± 29.9 ng m^−3^).

**Table 2:** Values of the measured concentrations of PM_2.5_ chemical components, as mean and standard deviation values of the whole study and of the spring and winter campaigns, separately. Bold values indicate statistically significant differences between seasons: *p < 0.005; **p ≤ 0.001

	Total		Spring		Winter	
	Mean	SD	Mean	SD	Mean	SD
ƩPAHs (ng m^−3^)	5.03	2.30	3.16**	0.53	6.58**	1.03
Ʃoxy-PAHs (ng m^−3^)	1.44	0.24	1.44	0.14	1.45	0.31
PM_2.5_ (μg m^−3^)	18.2	14.7	8.29**	3.28	26.7**	15.5
SO_4_^2−^ (μg m^−3^)	1.36	0.98	1.48	1.00	1.26	0.98
NO_3_^−^ (μg m^−3^)	4.85	6.43	0.48**	0.21	8.67**	6.82
NH_4_^+^ (μg m^−3^)	2.35	2.43	0.85**	0.41	3.70**	2.72
EC (μg m^−3^)	0.91	0.58	0.43**	0.06	1.31**	0.51
OC (μg m^−3^)	4.02	2.45	2.21**	0.63	5.51**	2.39
Levoglucosan (ng m^−3^)	251.2	317.7	10.6**	7.8	510.4**	279.9
Cl^−^ (μg m^−3^)	-	-	-	-	0.23	0.09
K^+^ (μg m^−3^)	-	-	-	-	0.31	0.21
Ca^2+^ (μg m^−3^)	-	-	-	-	0.9	0.4
As (ng m^−3^)	0.26	0.17	0.17*	0.07	0.35*	0.19
Fe (ng m^−3^)	57.1	29.9	49.0	19.1	65.2	40.7
V (ng m^−3^)	0.72	0.94	0.27*	0.14	1.17*	0.87
Zn (ng m^−3^)	15.4	12.3	6.9**	2.6	22.9**	12.6
Cd (ng m^−3^)	0.07	0.06	0.03**	0.01	0.11**	0.06
Pb (ng m^−3^)	2.67	2.08	1.29**	0.47	3.88**	2.20
Sn (ng m^−3^)	1.56	1.47	0.66**	0.22	2.34**	1.66
Sb (ng m^−3^)	0.52	0.20	0.41	0.09	0.58	0.22
La (ng m^−3^)	0.07	0.06	0.04*	0.02	0.11*	0.07
Mn (ng m^−3^)	-	-	1.45	0.33	-	-
OP_V_^DTT^ (nmol min^−1^ m^−3^)	0.21	0.21	0.08**	0.03	0.32**	0.15
OP_V_^AA^ (nmol min^−1^ m^−3^)	0.52	0.41	0.28**	0.21	0.72**	0.36
OP_m_^DTT^ (nmol min^−1^ μg^−1^)	0.017	0.031	0.004*	0.004	0.022*	0.022
OP_m_^AA^ (nmol min^−1^ μg^−1^)	0.034	0.031	0.033	0.023	0.035	0.037

The total ΣPAHs concentration was related with PM_2.5_ components that are markers of specific emission sources, in order to give insight into PAH source apportionment. The results of the conducted Kendall rank correlation analysis are given in Table 3 (statistically significant correlations are indicated by bold values). Investigating the whole year dataset, we can observe that the total ΣPAHs resulted significantly associated with most of the PM_2.5_ components, other than with the PM_2.5_ mass, namely with EC, OC, NO_3_^−^ ion, levoglucosan, Zn, Cd, Pb, Sn, and La metals at very significant correlation level (*p* < 0.01) and with NH_4_^+^ ion, As, V, and Sb at *p* < 0.05 level. Among these components, the correlation with levoglucosan and organic carbonaceous component confirmed that biomass burning was the main source of PM_2.5_-bound PAHs, as these species are specific tracers of such an emission source. This played a dominant contribution during the cold season, when wood fuel is widely used for residential heating, as showed by the high levoglucosan level (510.4 ± 279.9 ng m^−3^, Table 2) (Hakimzadeh et al. 2020; Nalin et al. 2018; Pietrogrande et al. 2011, 2016). In addition, the significant rank correlations with EC and some metals supported the concomitant contribution from exhaust and non-exhaust emissions from on-road vehicles, as Zn, Cd, Pb, Sn, La, and Sb have been found related with vehicle emission, typically in urban areas (Hao et al. 2018; Lin et al. 2019; Riccio et al. 2016). It is noteworthy that most correlations between concentrations of ΣPAHs and chemical species were lost when spring and winter data were investigated, separately; i.e., ΣPAHs was found significantly associated (*p* < 0.01) only with EC, Sn, and La during the winter campaign.

**Table 3:** Correlation of the concentrations of ΣPAHs and Σoxy-PAHs with those of other PM_2.5_ components: Kendall rank correlation computed on the whole dataset and on spring and winter data, separately. Bold values indicate statistically significant correlations: * significant correlations (*p* < 0.05); ** very significant correlation (*p* < 0.01)

	Total		Spring		Winter	
	ΣPAHs	Σoxy-PAHs	ΣPAHs	Σoxy-PAHs	ΣPAHs	Σoxy-PAHs
ΣPAHs	1.00	0.17	1.00	0.50**	1.00	0.72**
Σoxy-PAHs	0.17	1.00	0.50**	1.00	0.72**	1.00
PM_2.5_	0.46**	− 0.22	0.01	− 0.15	0.21	0.06
EC	0.68**	− 0.06	0.19	− 0.15	0.46**	0.39*
OC	0.55**	− 0.16	0.00	− 0.11	0.28	0.11
SO_4_^2−^	− 0.01	0.03	0.18	− 0.02	0.11	0.04
NO_3_^−^	0.48**	− 0.23	0.01	− 0.17	0.03	− 0.13
NH_4_^+^	0.42*	− 0.19	0.12	− 0.10	0.04	− 0.11
Levoglucosan	0.67**	− 0.04	0.39*	0.30	0.39*	0.09
K^+^	-	-	-	-	0.10	− 0.01
Cl^−^	-	-	-	-	0.25	0.07
Ca^2+^	-	-	-	-	− 0.31	− 0.33
As	0.35*	− 0.13	− 0.11	− 0.04	0.20	0.06
Fe	**0.16**	− 0.05	− 0.27	− 0.27	0.38*	0.37*
V	− 0.35*	− 0.09	− 0.04	− 0.25	− 0.02	− 0.15
Zn	0.60**	− 0.10	0.19	0.13	0.30	0.18
Cd	0.52**	− 0.07	0.22	0.15	0.21	0.10
Pb	0.48**	− 0.07	0.14	0.06	0.26	0.12
Sn	0.51**	− 0.09	− 0.19	− 0.10	0.47**	0.35
Sb	0.36*	0.03	0.49	0.15	0.13	0.20
La	0.48**	− 0.14	− 0.16	− 0.43**	0.49**	0.52**
Mn	-	-	− 0.18	− 0.29	-	-

### Quinone concentrations: seasonal variations and distribution profiles

Five quinones were identified in the investigated PM_2.5_ samples. The mean concentration values were computed for the spring and winter campaigns, separately (Table 1). Their individual concentrations ranged from 0.17 to 0.48 ng m^−3^, with similar values in the two seasons, in contrast with PAH levels, that showed a strong seasonality (Table 1). The detected oxy-PAHs contained 2 or 3 benzene rings and 1 or 2 oxygen groups. Their concentration profiles were dominated by 3 benzene rings species, i.e., 5,12-NQ (0.48 ± 0.08 ng m^−3^) and 7,12-BAQ (0.42 ± 0.02 ng m^−3^), followed by 2 benzene rings congeners, i.e., 9,10-AQ (0.27 ± 0.03 ng m^−3^), 9-FLO (0.24 ± 0.01 ng m^−3^), and XAN (0.22 ± 0.02 ng m^−3^). Widely scattered data are reported in literature (Table 4) that may arise from strongly differing degrees of local pollution. Inside such a variation, the concentrations measured in our work (Table 1) appear broadly consistent with those determined in other urban background sites in Europe (Andreou and Rapsomanikis 2009; Delgado-Saborit et al. 2013; Pietrogrande et al. 2011) and the USA (Delhomme et al. 2008; Kramer et al. 2020).

**Table 4:** Data from the literature of the concentrations of each measured oxy-PAH

Compound	Concentration (ng m^−3^)	Location	Season	Reference
9-FLO	3.577 1.04 0.431 1.14 1.13 0.769 0.028 4.79 ± 2.65 5.31 ± 1.48 8.30 ± 3.75 4.82 ± 1.94 5.04 ± 3.86 10.3 ± 2.40	Marseilles, France Oporto, Portugal Oporto, Portugal Florence, Italy Florence, Italy Athens, Greece Athens, Greece Beijing, China Tianjin, China Shijiazhuang, China Hengshui, China Jinan, China Jinan, China	S W S W S W S W W W W S W	Albinet et al. (2007) Alves et al. (2017) Alves et al. (2017) Alves et al. (2017) Alves et al. (2017) Alves et al. (2017) Alves et al. (2017) Niu et al. (2017) Niu et al. (2017) Niu et al. (2017) Niu et al. (2017) Li et al. (2019) Li et al. (2019)
XAN	0.25 0.27 ± 0.31 0.36 ± 1.02 0.30 ± 0.25 0.06 ± 0.05 0.7489	Tempe, USA Athens, Greece Athens, Greece Augsburg, Germany Augsburg, Germany Ohio, USA	Sp S W W S S	Delhomme et al. (2008) Andreou and Rapsomanikis (2009) Andreou and Rapsomanikis (2009) Pietrogrande et al. (2011) Pietrogrande et al (2011) Kramer et al. (2020)
9,10-AQ	0.21 ± 0.22 0.47 ± 0.46 1.63 0.037 0.033 0.49 ± 0.12 1.38 ± 0.36	California, USA California, USA Tempe, USA Weybourne, UK Weybourne, UK Jinan, China Jinan, China	S W Sp W S S W	Chung et al. (2006) Chung et al. (2006) Delhomme et al. (2008) Alam et al. (2014) Alam et al. (2014) Li et al. (2019) Li et al. (2019)
7,12-BAQ	1.0 ± 0.71 0.12 0.35 0.19 ± 0.19 0.24 ± 0.56 0.20 ± 0.13 0.02 ± 0.02 0.50 0.015 0.011 0.978 0.331 0.112 0.227 4.88 ± 4.15 4.74 ± 2.35 10.58 ± 5.61 7.62 ± 3.75 0.21 ± 0.13 0.17 ± 0.05	California, USA Marseilles, France Tempe, USA Athens, Greece Athens, Greece Augsburg, Germany Augsburg, Germany Birmingham, UK Weybourne, UK Weybourne, UK Oporto, Portugal Oporto, Portugal Florence, Italy Athens, Greece Beijing, China Tianjin, China Shijiazhuang, China Hengshui, China Jinan, China Jinan, China	W S Sp S W W S W W S W S W W W W W W S W	Chung et al. (2006) Albinet et al. (2007) Delhomme et al. (2008) Andreou and Rapsomanikis (2009) Andreou and Rapsomanikis (2009) Pietrogrande et al. (2011) Pietrogrande et al. (2011) Delgado-Saborit et al. (2013) Alam et al. (2014) Alam et al. (2014) Alves et al. (2017) Alves et al. (2017) Alves et al. (2017) Alves et al. (2017) Niu et al. (2017) Niu et al. (2017) Niu et al. (2017) Niu et al. (2017) Li et al. (2019) Li et al. (2019)
5,12-NQ	0.04 ± 0.03 0.93 ± 1.6 4.5 ± 9.5 0.016 0.011 0.452 0.321 0.61 0.551 1,47 ± 1,28 2.08 ± 1.72 3.89 ± 2.61 4.18 ± 2.61 0.4688	Santiago, Chile California, USA California, USA Weybourne, UK Weybourne, UK Oporto, Portugal Oporto, Portugal Florence, Italy Athens, Greece Beijing, China Tianjin, China Shijiazhuang, China Hengshui, China Ohio, USA	S W S W S W S W W W W W W S	Tsapakis et al. (2002) Chung et al. (2006) Chung et al. (2006) Alam et al. (2014) Alam et al. (2014) Alves et al. (2017) Alves et al. (2017) Alves et al. (2017) Alves et al. (2017) Niu et al. (2017) Niu et al. (2017) Niu et al. (2017) Niu et al. (2017) Kramer et al. (2020)

*S* summer, *W* winter, *Sp* spring

To give information on the oxy-PAHs origin, the intercorrelation between quinones and parent PAHs concentrations were investigated, since they may discriminate between primary emission from combustion processes or secondary production from atmospheric reactions (Alves et al. 2017; Harrison et al. 2016; Niu et al. 2017; Walgraeve et al. 2010). The correlation between Ʃoxy-PAHs and ƩPAHs resulted not significant when the whole dataset was analyzed while very significant (Kendall rank correlation at *p* < 0.01) when spring and winter data were investigated separately (Table 3). Since this result may suggest different origin of oxy-PAHs in the two seasons, the correlations between the individual PAHs and oxy-derivatives were investigated in detail by separating spring and winter data (Kendall’s coefficients in Table 5). In winter, among the investigated quinones, the lightest 9-FLO and XAN and the heaviest 5,12-NQ resulted well positively correlated (most at *p* < 0.01) with nearly all the investigated PAHs. Such results potentially suggest common sources for parent and derivative PAHs, as mainly co-emitted from the same primary combustion sources. They may be likely dominated by the contribution of biomass burning emission, as supported by the strong correlation of ΣPAHs with levoglucosan (Table 3), and the high winter concentrations of individual PAHs and oxy-PAHs, likely associated with the increased primary emission from residential wood combustion for household heating (Alves et al. 2017; Amato et al. 2016; Belis et al. 2011), as previously found in the investigated region (Pietrogrande et al. 2016; Ricciardelli et al. 2017).

**Table 5:** Correlation between concentrations of ΣPAHs and Σoxy-PAHs: Kendall rank correlation computed on the winter and spring data, separately. Bold values indicate statistically significant correlations: * significant correlations (*p* < 0.05); ** very significant correlation (*p* < 0.01)

Winter	*9-FLO*	*XAN*	*9,10-AQ*	*7,12-BAQ*	*5,12-NQ*
ACENY	0.44	0.30	0.09	− 0.07	− 0.07
ACE	0.58*	0.52*	0.45	− 0.07	− 0.05
FLU	0.79**	0.75**	0.52*	0.30	0.32
PHE	0.73**	0.62**	0.43	0.30	0.28
ANT	0.86**	0.77**	0.29	− 0.31	− 0.41
FLUA	0.93**	0.77**	0.19	0.02	0.05
PYR	0.94**	0.79**	0.24	0.00	0.03
BaA	0.11	0.18	− 0.09	0.94**	0.97**
CRY	− 0.09	− 0.06	− 0.34	0.28	0.33
BbF	0.68**	0.46	0.03	0.05	0.02
BkF	0.70**	0.48	0.04	0.03	0.01
BaP	0.61**	0.51*	0.23	0.04	0.02
InP	0.58*	0.33	0.07	− 0.01	− 0.06
DbA	0.14	− 0.23	− 0.48	0.11	− 0.05
BghiP	0.51*	0.30	0.09	− 0.03	− 0.03
Spring	***9-FLO***	***XAN***	***9,10-AQ***	***7,12-BAQ***	***5,12-NQ***
FLU	0.50	0.41	0.36	− 0.12	0.02
PHE	0.26	0.24	0.28	0.81**	0.71**
ANT	0.60*	0.21	0.44	0.22	0.25
FLUA	0.50	0.23	0.75**	0.29	0.23
PYR	0.30	-0.08	0.59*	− 0.24	− 0.10
CRY	0.39	0.36	0.92**	0.14	0.02
BbF	0.53*	0.45	0.16	− 0.05	0.09
BkF	− 0.24	− 0.31	− 0.32	− 0.32	− 0.21
BaP	0.46	0.17	0.07	− 0.41	− 0.13
BghiP	0.22	0.20	0.43	− 0.03	− 0.18

Otherwise, in spring, only 9,10-AQ resulted positively correlated (most at *p* < 0.05) with some investigated PAHs. This suggests that the measured quinones were mainly generated from complex atmosphere oxidation reactions, also involving multiple oxidation steps including very reactive intermediates, so that the produced quinones have lost the link with the profile of the parent PAHs. This also motivates the lack of light PAHs—ACENY and ACE and even the four rings BaA—in the PM-bound phase, as they are depleted by heterogeneous reactions with atmospheric oxidants, such as hydroxyl radical, ozone, and NOx (Keyte et al. 2013; Li et al. 2019). These results are confirmed by studies based on laboratory and road tunnel experiments (Riccio et al. 2016; Ringuet et al. 2012).

Thus, the ratios between oxygenated derivatives and their parent PAHs were investigated in detail to gain a deeper insight into these atmospheric processes. Overall, the total Σoxy-PAHs/ΣPAHs ratio was nearly double in spring, 0.46 ± 0.11, compared with winter, 0.23 ± 0.06, suggesting that the photochemical and thermal degradation of the individual PAHs were dominating in the hot season. This is consistent with the higher oxidative capacity of the atmosphere in spring, mainly due to the higher temperature and stronger solar radiation, as observed in previous studies (Albinet et al. 2007; Alves et al. 2017; Harrison et al. 2016; Pietrogrande et al. 2011; Walgraeve et al. 2010). This result was also confirmed by investigating some specific quinone/parent PAH ratios. They were found nearly double in spring compared with winter: the mean 9-FLO:FLU ratio was 0.4 ± 0.1 in winter and 0.8 ± 0.4 in spring, and 9,10-AQ:ANT value was 0.5 ± 0.2 in winter and 0.9 ± 0.1 in spring. In general, the measured values appear broadly consistent with the literature data concerning less aged air in urban area, as found in mainland Europe and in the UK (Alam et al. 2014; Harrison et al. 2016; Keyte et al. 2013; Walgraeve et al. 2010).

### OP responses from the DTT and AA assays

**Fig. 2:**
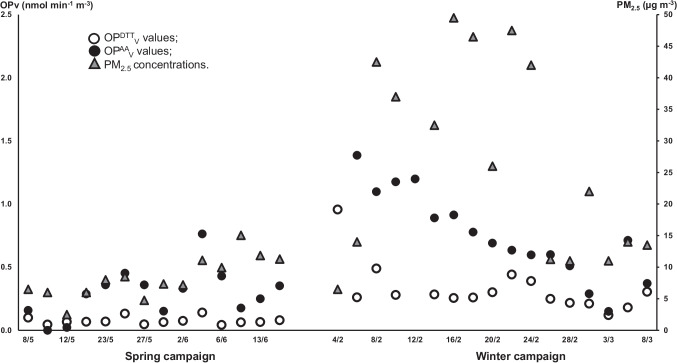
Day evolution of the volume-based OP^DTT^_V_ and OP^AA^_V_ responses (left scale) and PM_2.5_ concentrations (right scale) during the two campaigns: spring, from 8 May to 17 June 2019, and winter, from 4 February to 9 March 2020. Black points: OP^DTT^_V_ values; empty points: OP^AA^_V_ values; gray triangles: PM_2.5_ concentrations

The redox activity of the PM_2.5_ samples was measured by using the DTT and AA acellular assays. The volume-based OP_V_^DTT^ and OP_V_^AA^ responses were computed, as a relevant metric of human exposition concern. Their day evolution during the two campaigns is reported in Fig. 2 (left scale), together with that of PM_2.5_ mass concentrations (right scale). The average values were computed on the whole dataset and on the spring and winter data, separately (Table 2). A deep insight into the figure illustrates fast variations from day to day, which may be related to changes in the PM_2.5_ concentration related to sources or meteorological conditions or/and to the PM_2.5_ chemistry or composition. In particular, during spring, the values of OP_V_^DTT^ (empty points) were sometimes close to 0, whereas OP_V_^AA^ (black points) showed larger values. Moreover, the OP_V_^AA^ responses varied more widely scattered than the OP_V_^DTT^ values, especially during winter. The clear seasonality of both OP_V_^DTT^ and OP_V_^AA^ is described by a significant increase of the OP_V_ responses in winter compared with spring, i.e., from 0.08 ± 0.03 to 0.32 ± 0.15 nmol min^−1^ m^−3^ for OP_V_^DTT^ and from 0.28 ± 0.21 to 0.72 ± 0.36 nmol min^−1^ m^−3^ for OP_V_^AA^ (significant difference at *p* < 0.05 signed by * in Table 2). Such a seasonality, which closely follows that of the PM_2.5_ mass, has been previously found in Po Valley and in other European cities (Hakimzadeh et al. 2020; Paraskevopoulou et al. 2019 and reference therein; Pietrogrande et al. 2019b). It has been explained by the increased emissions of redox-active compounds from anthropogenic sources, mainly biomass burning, combined with pollutant accumulation due to the stagnant atmospheric conditions (Belis et al. 2011; Masiol et al. 2013; Pietrogrande et al. 2016; Ricciardelli et al. 2017). Figure 1 clearly shows that the OP_V_ responses from the DTT (empty points) were always lower compared with those from the AA assay (black points), with total average of 0.21 ± 0.21 nmol min^−1^ m^−3^ and 0.52 ± 0.41 nmol min^−1^ m^−3^, respectively. This trend has been related to the different sensitivity of the two assays towards the same redox-active PM components driving OP responses, as found in other studies (i.e., Crobeddu et al. 2017; Fang et al. 2016; Hellack et al. 2017; Molina et al. 2020; Simonetti et al. 2018). Although such differences, the measured OP_V_^DTT^ and OP_V_^AA^ responses were found very significantly intercorrelated (*p* < 0.01, bold values in Table 6), consistently with previous studies (Bates et al. 2019; Calas et al. 2019; Pietrogrande et al. 2019b).

**Table 6:** Correlation between OP_V_^DTT^ and OP_V_^AA^ responses and concentrations of ΣPAHs and Σoxy-PAHs and other PM_2.5_ components: Kendall rank correlation computed on the whole dataset and on spring and winter data, separately. Bold values indicate statistically significant correlations: * significant correlations (*p* < 0.05); ** very significant correlation (*p* < 0.01)

	Total		Spring		Winter	
	OP_V_^DTT^	OP_V_^AA^	OP_V_^DTT^	OP_V_^AA^	OP_V_^DTT^	OP_V_^AA^
OP_V_^DTT^	1.00	0.51**	1.00	0.23	1.00	0.23
OP_V_^AA^	0.51**	1.00	0.23	1.00	0.23	1.00
ƩPAHs	0.60**	0.54**	0.07	0.00	0.47*	0.39*
Ʃoxy-PAHs	− 0.11	0.01	− 0.01	0.11	0.49*	0.37
PM_2.5_	0.51**	0.47**	0.14	0.20	0.23	0.27
EC	0.60**	0.53**	0.06	-0.05	0.24	0.54**
OC	0.62**	0.52**	0.20	0.14	0.39*	0.32
SO_4_^2−^	− 0.03	0.14	0.20	0.32	0.09	0.17
NO_3_^−^	0.56**	0.38**	0.00	0.00	0.19	0.05
NH_4_^+^	0.47**	0.43**	0.12	0.47*	0.14	− 0.02
Levoglucosan	0.64**	0.45**	0.18	− 0.24	0.26	0.12
K^+^	-	-	-	-	0.26	0.06
Cl^−^	-	-	-	-	− 0.11	0.28
Ca^2+^	-	-	-	-	− 0.24	− 0.02
As	0.47**	0.41**	0.33	0.19	0.28	0.29
Fe	0.19	0.37*	0.29	0.01	0.03	0.67**
V	− 0.29	− 0.12	− 0.04	0.19	0.25	0.34
Zn	0.68**	0.46**	0.50*	− 0.11	0.31	0.44*
Cd	0.54**	0.53**	− 0.07	0.43	0.30	0.39*
Pb	0.53**	0.45**	0.17	0.27	0.37	0.23
Sn	0.59**	0.52**	0.36	0.16	0.27	0.55**
Sb	0.38*	0.41*	0.00	0.09	0.29	0.32
La	0.62**	0.50**	0.22	− 0.10	0.52**	0.46*
Mn	-	-	0.32	− 0.08	-	-

The measured OP_V_^DTT^ data are within the range reported for other urban sites in Italy, as recently reviewed by the authors (Pietrogrande et al. 2019b), even if lower than the values from previous studies in the same area (1.15 nmol min^−1^ m^−3^) and in Milan (~ 3.38 nmol/min^−1^ m^−3^) (Hakimzadeh et al. 2020). Otherwise, the measured OP_V_^AA^ data are very close to those previous found in the same area (0.7 nmol min^−1^ m^−3^, Pietrogrande et al. 2019a) but higher than those reported for other urban sites in Italy (Pietrogrande et al. 2019b; Simonetti et al. 2018).

Both OP responses were also expressed as mass-related OP_m_ values, in order to assess the PM_2.5_ intrinsic redox toxicity. The obtained OP_m_^DTT^ responses were in general very low (mean value: 0.017 ± 0.031 nmol min^−1^ μg^−1^), while the OP_m_^AA^ responses were nearly double (mean values: 0.034 ± 0.031 nmol min^−1^ μg^−1^) (Table 2). This observation further confirms that the investigated samples contained high concentrations of redox-active components more reactive towards the AA than the DTT assays (Crobeddu et al. 2017; Fang et al. 2016; Hellack et al. 2017; Molina et al. 2020).

### Association of the measured OP_V_ values with PAH and oxy-PAH concentrations

The potential role of PAHs and their oxo-derivatives in affecting PM oxidative properties was investigated in detail by conducting Kendall rank correlation between the PM_2.5_ OP responses and concentrations of ƩPAHs, Ʃoxy-PAHs, and other chemical components (Kendall’s τ coefficient given in Table 6). It is noteworthy that such associations may be affected by some biases, since the various parameters were measured using different analytical protocols and thus may represent different fractions of the PM_2.5_ components. Most papers on the topic carried out the OP assays on water-soluble PM fraction, extracted with water or aqueous buffers, to obtain the bio-available components, in order to simulate PM/cells interactions. A recent authors’ paper showed that both the measured OP^DTT^ and OP^AA^ responses varied by changing the extraction solvents, with phosphate buffer generating the highest values and methanol the lowest (Pietrogrande et al. 2021). Therefore, the phosphate buffer has been proved as the most useful solvent for OP assay, providing the most sensible OP measures. Other chemical components are commonly analyzed using different PM extraction and pre-treatment procedures, by selecting the proper conditions assuring the most accurate results for each analyte class, in order to represent the real PM chemical composition. In the common procedures, the polar organic compounds, such as PAHs and oxy-PAHs, are extracted with solvent mixture with varying polarity, inorganic ions and anhydrosugars are extracted with water/buffer, and total metals are analyzed after PM acid mineralization. Although the different extraction yields, the various solvents were found to produce intercorrelated OP values for real PM_2.5_ samples as well as for standard mixtures of transition metals and quinones (Hellack et al. 2017; Pietrogrande et al. 2021). This suggests that they can be useful for investigating the association between PM OP and chemical composition, as also confirmed by the significant correlations widely reported in literature (i.e., Bates et al. 2019; Calas et al. 2019; Hakimzadeh et al. 2020; Paraskevopoulou et al. 2019; Pietrogrande et al. 2019b; Wang et al. 2018).

The total ƩPAHs concentration resulted significantly (*p* < 0.01) correlated with both OP_V_^DDT^ and OP_V_^AA^ responses of the whole dataset and only with OP_V_^DDT^ values (*p* < 0.05) of winter data, separately (Table 6), thus suggesting that these compounds are involved in inducing PM oxidative property. Although this result has been already found in other papers, it is difficult to be explained, as the PAH congeners have been found inactive on certain biological end points, including ROS generation (Jin et al. 2019; Molina et al. 2020; Tuet et al. 2019). Most papers attributed such an association to the co-linearity of these components with truly active PAH derivatives, which originated from the same combustion sources or generated by atmosphere oxidation processes of the parent PAHs. Thus, total ƩPAHs concentration can be considered a general descriptor of the complex mixture of the PM organic aromatic components, including truly redox-active compounds, such as quinone-like compounds (Kramer et al. 2020; Molina et al. 2020; Pietrogrande et al. 2019b; Pirhadi et al. 2020; Wang et al. 2018). This is supported by the significant correlation separately found between ƩPAHs and Ʃoxy-PAHs concentrations measured in each season, characterized by specific oxy-PAH origin and processes (Tables 3). This explanation is consistent with the finding that the OP_V_ responses, as well as ƩPAHs, were significantly correlated with markers of the main PAH emission sources, namely with levoglucosan, tracer of biomass burning, and with EC and metals related with vehicular emissions (Zn, Cd, Pb, Sn, Sb, and La, mostly at *p* < 0.01) (Table 6). This last association underlines that the PM-bound metals significantly contribute to the ROS-generation potential, as observed in previous studies (Bates et al. 2019; Charrier and Anastasio 2015; Paraskevopoulou et al. 2019). This likely motivates the measured higher OP_V_^AA^ responses compared with OP_V_^DTT^ values, since the investigate samples showed high levels of those metals that are very reactive to the AA assay, in particular Fe and Zn but also Pb and Sn (Table 2). Such a pattern has been generally found in the urban sites strongly impacted by traffic emissions (Fang et al. 2016; Lyu et al. 2018; Jin et al. 2019; Pietrogrande et al. 2019b; Simonetti et al. 2018).

The result that the OP_V_^DTT^ responses are significantly (*p* < 0.05) associated with total Ʃoxy-PAHs concentration only for the winter data (Table 6) can be explained with the dominating contribution of the biomass burning for domestic heating in the cold period. This is supported by the correlation of individual quinones, as well as Ʃoxy-PAHs, with individual and total PAHs, and also with levoglucosan (Table 2). In addition, the redox-active quinones can also be formed from secondary oxidation of freshly emitted PAHs in smoke particles (Li et al. 2019; Lyu et al. 2018; Jiang et al. 2016; Wang et al. 2018). The significant role of secondary organic aerosol in driving OP of aged soot particles is supported by the association of OP_V_^DTT^ with OC (*p* < 0.05), as marker of secondary organics. This is consistent with the results from laboratory experiments and ambient studies that showed the dominant contribution of the quinone-like components emitted from biomass burning to aerosol OP, mainly during the cold period, both considering fresh biomass burning emissions and secondarily oxidized organic components (Hakimzadeh et al. 2020; Jin et al. 2019; Molina et al. 2020; Paraskevopoulou et al. 2019; Pietrogrande et al. 2019b; Tuet et al. 2019).

Finally, both OP_V_^DDT^ and OP_V_^AA^ responses of the whole dataset were very significantly (*p* < 0.01) correlated with the carbonaceous fraction OC and with secondary ions NO_3_^−^ and NH^4+^, supporting that the aged organics originated from the oxidation of primary organic components are important contributors to the PM-induced oxidative stress (Belis et al. 2011; Hakimzadeh et al. 2020; Pietrogrande et al. 2016; Ricciardelli et al. 2017).

It should, however, be noted that caution must be exercised when interpreting correlation results, as the relationship between correlation and causation is not simple and some results may be a likely consequence of covariance between the variables, such as metals and quinones. Further, the correlation results may be potentially affected by other PM components not identified in this study that may induce ROS production. In particular, it must be underlined that in the investigated samples, we did not detect the quinones frequently identified as drivers of PM OP, i.e., 1,2-naphthoquinone, 1,4-naphthoquinone, and 9,10-phenanthrenequinone (Charrier and Anastasio 2015).

## Conclusions

Fifteen PM_2.5_-bond PAHs and five quinones were quantified in the atmosphere of a Po Valley city and their distribution profiles investigated. Total PAHs showed nearly double concentrations in winter compared with spring, in contrast with quinones, which exhibited constant levels in both seasons. This may be explained by differences in oxy-PAHs origin, as they are mainly emitted from primary combustion sources in winter—vehicle exhaust and biomass burning—and generated from photochemical degradation of parent PAHs in spring.

The results of this study revealed that total PAH concentration is significantly associated with PM_2.5_ oxidative properties, as a general descriptor of the redox-active organic components of ambient aerosol, in particular, quinone-like compounds directly co-emitted or secondarily produced by oxidation of parent PAHs from biomass combustion or vehicle traffic. In winter, the total oxy-PAHs were found significantly correlated with the OP_V_^DTT^ responses, mainly associated with combustion particles from wood burning.

Overall, it is difficult to apportion the PM oxidative activity to specific chemical compounds from the different origins, as it is given by the combination of the specific reactivity of each individual component with its concentration level in PM. This highlights the need of further work for improving analytical methods, allowing for more precise characterization of redox-active PAH derivatives present in PM_2.5_ particles. A better understanding of the formation pathways and the environment fate of oxy-PAHs and different contributors to ROS generation will help to guide strategies for targeted mitigation of the atmospheric oxidation potential in order to reduce the great disease stress caused by exposure to air pollution.

## Data Availability

The datasets used and analyzed during the current study are available from the corresponding author on reasonable request.
